# Genome-wide analysis of G-quadruplexes in herpesvirus genomes

**DOI:** 10.1186/s12864-016-3282-1

**Published:** 2016-11-21

**Authors:** Banhi Biswas, Manish Kandpal, Utkarsh Kumar Jauhari, Perumal Vivekanandan

**Affiliations:** 1 Kusuma School of Biological Sciences, Indian Institute of Technology Delhi, New Delhi, 110016 India; 2 Department of Civil Engineering, Indian Institute of Technology Delhi, New Delhi, 110016 India

**Keywords:** G-quadruplexes, Herpesviruses, Repeat region, Regulatory regions, Temporal regulation

## Abstract

**Background:**

G-quadruplexes are increasingly recognized as regulatory elements in human, animal, bacterial and plant genomes. The presence and function of G-quadruplexes are not well studied among herpesviruses; in particular, there are no systematic genome-wide analysis of these important secondary structures in herpesvirus genomes.

**Results:**

We performed genome-wide analysis of putative quadruplex sequences (PQS) in human herpesviruses. We found unusually high PQS densities among human herpesviruses. PQS are enriched in the repeat regions and regulatory regions of human herpesviruses. Interestingly, PQS densities are higher in regulatory regions of immediate early genes compared to early and late genes in most herpesviruses. In addition, the majority of genes functionally conserved across human herpesviruses contain one or more PQS within the regulatory regions. We also describe the existence of unique intramolecular PQS repeats or repetitive G-quadruplex motifs in herpesviruses. Functional studies confirm a role for G-quadruplexes in regulating the gene expression of human herpesviruses.

**Conclusion:**

The pervasiveness of PQS, their enrichment and conservation at specific genomic locations suggest that these structural entities may represent a novel class of functional elements in herpesviruses. Our findings provide the necessary framework for studies on the biological role of G-quadruplexes in herpesviruses.

**Electronic supplementary material:**

The online version of this article (doi:10.1186/s12864-016-3282-1) contains supplementary material, which is available to authorized users.

## Background

Recently, nucleic acid secondary structures, G-quadruplexes in particular, have received much attention. G-quadruplexes are non-canonical nucleic acid secondary structures that are formed from G-rich sequences. These sequences consist of four stretches of G residues (each stretch with two or more G residues) interspersed by sequences of variable composition that form the loops. The formation of G-quadruplexes is induced and stabilized by monovalent cations like potassium and sodium. The presence of G-quadruplexes was first reported in telomeres and subsequently in the promoter region of several genes, 5′UTR (untranslated regions) and 3′UTRs [1–6]. G-quadruplexes function as regulatory elements and can influence key biological processes including transcription [3], translation [4] and splicing [7]. Recently, G-quadruplexes were also reported to be enriched at certain positions of eukaryotic retrotransposons, which correspond to regulatory regions of genes and viruses [8, 9]. Genome-wide studies on human, animal and bacterial genomes demonstrate the presence of G-quadruplexes in regulatory regions proximal to the transcription start sites (TSS) [10–12]. Studies on G-quadruplexes in virus genomes are limited. A few studies show functional roles of selected RNA or DNA G-quadruplex motifs present in the virus genome in vitro [13–17] or describe the genomic location of G-quadruplexes in virus genomes [18].

Herpesviruses are ubiquitous large DNA viruses that are amongst the best known host-adapted viruses. We chose to investigate the genome-wide distribution of G-quadruplexes and their potential as novel regulatory elements among herpesviruses since they have long term relationship with the host [19] and G-quadruplexes are major regulators of gene expression in human; it is possible that human herpesviruses may exploit host regulatory mechanisms via G-quadruplexes in their genome. In addition, transcription in large DNA viruses is completely dependent on the host transcription machinery [20] and the mimicking of host-regulatory elements is an essential part of virus evolution. Herpesviruses are known to mimic host proteins for their own benefit. Studies have shown over 10% of herpesvirus genes are homologues of the host genes [21]. In addition to their ability to mimic host proteins [21], viruses may also mimic host promoters [20]. Given that G-quadruplexes are established transcriptional regulatory elements in human [3, 22, 23] it is possible that the simulation of structural DNA regulatory elements of the host by the virus may help complete the virus life cycle.

Herpesviruses have a linear double-stranded DNA (ds-DNA) genome. The genomes of human herpesviruses vary between 125–235 Kb in size. Based on differences in biological properties human herpesviruses are classified into 3 subfamilies namely alphaherpesvirus (human herpesvirus-1, –2, and -3), betaherpesviruses (human herpesvirus-5, –6a, –6b, –7) and gammaherpesviruses (human herpesvirus-4, –8) [24].

In this work we have identified G-quadruplexes within herpesvirus genomes as novel regulators of herpesvirus gene expression. Our results demonstrate the association of PQS with unique genomic features including regulatory regions, repeat regions and immediate early genes. We also describe the presence of unique intramolecular putative quadruplex sequences (PQS) repeats or repetitive intramolecular G-quadruplexes in herpesviruses. Importantly, our results suggest yet unknown putative roles for G-quadruplexes in herpesvirus genomes.

## Results and discussion

### Herpesviruses genomes have unusually high PQS densities

**Fig. 1:**
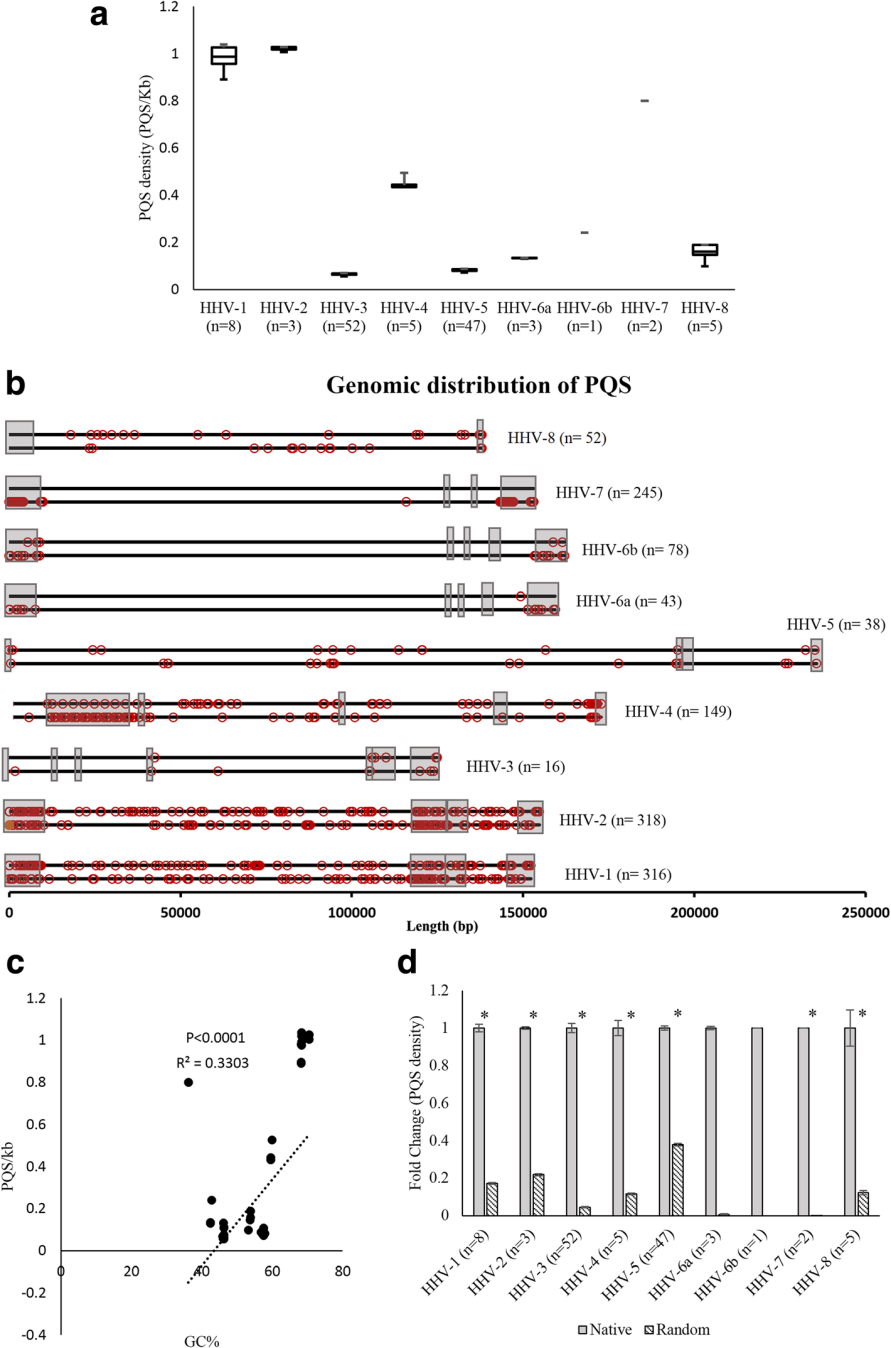
Genome-wide PQS densities and distribution of PQS. **a** Box plots showing average PQS densities (PQS/Kb) in full-length genomes of herpesviruses (HHV-1 to HHV-8; both strands for each virus). All available full-length sequences were analysed: HHV-1 (*n* = 8), HHV-2 (*n* = 3), HHV-3 (*n* = 52), HHV-4 (*n* = 5), HHV-5 (*n* = 47), HHV-6A (*n* = 3), HHV-6B (*n* = 1), HHV-7 (*n* = 2), HHV-8 (*n* = 5); please see Additional file 1 for accession numbers. The upper quartile in the box plot denotes the 75^th^ percentile, the lower quartile denotes 25^th^ percentile the upper and the lower bar denotes the maximum and minimum values and the central horizontal line denotes the median. **b** Schematic representation of the distribution of PQS in human herpesviruses. The genomes are drawn to scale. The black lines represent the two strands of the genomes. For each virus the upper black line represents the strand of DNA with 5′ to 3′ orientation (left to right) and the lower line represents the strand of DNA with 3′ to 5′ orientation (left to right). The red circles represent the genomic positions of PQS. The grey boxes represent the repeat regions in the genome. The total number of PQS present in a virus genome is shown in parentheses. Sequences used for creating the plot are HHV-1[Genbank*:*NC_001806], HHV-2[Genbank*:*NC_001798], HHV-3[Genbank*:*NC_001348], HHV-4 [Genbank*:*NC_007605], HHV-5[Genbank*:*NC_006273], HHV-6a[Genbank*:*NC_001664], HHV-6b[Genbank*:*NC_000898] HHV-7[Genbank*:*NC_001716] HHV-8[Genbank*:*NC_009333]. **c** Scatter plot showing correlation between GC% (x-axis) and PQS density (PQS/Kb) (y-axis) in herpesvirus genomes (*P* < 0.0001). **d** Native full length herpesvirus sequences (*n* = 126) are enriched for PQS compared to the randomized full length sequences (*n* = 630; five randomized sequences were analyzed for each native sequence). *denotes *P* < 0.001

We found an unusually high number of PQS in the genomes of human herpesviruses. Human herpesvirus 2 (HHV-2) had the highest number of PQS (*n* = 318) among human herpesviruses. The average number of PQS in human herpesvirus genomes ranges from 14 to 318. The distribution of PQS densities among all available sequences (Additional file 1: Table S1) of human herpesviruses is shown in Fig. 1a. The genomic locations of PQS within human herpesviruses are schematically represented in Fig. 1b. To analyze whether the presence of high number of PQS in herpesvirus genome is a result of biased nucleotide composition (high GC% in some of the herpesviruses, Fig. 1c) we performed a permutation test. Randomization of sequences was carried out as described in methods section. Native herpesvirus genomes had nearly 10 fold higher PQS densities compared to the randomized sequences having same mononucleotide composition as native sequences (Fig. 1d). In order to further vindicate that the high PQS densitites in human herpesvirus genomes are not a random phenomenon we randomized sequences in a sliding window of 40bp and mapped the PQS. The randomization of sequences in using a sliding window approach reconfirmed that PQS densities in native full length sequences (divided into 40bp sliding windows) were higher for most herpesviruses compared to randomized sequences (Additional file 2: Figure S1a). HHV-5 was an exception to this rule, with higher number of PQS in the randomized sequences than in the native sequences. These findings clearly show that the high PQS densities among herpes viruses is not a random phenomenon.

The human genome has a PQS density of 0.13/Kb [10] and the highest PQS density reported in literature for any organism is 0.19/Kb, for the mouse genome [25]. Interestingly, in our study we found PQS densities as high as 1.037/Kb (Fig. 1a) among herpesviruses, which is more than 7 fold higher than the PQS density reported for the human genome. The PQS densities of HHV-1 and HHV-2 (Fig. 1a) are the highest reported for any genome. The high PQS densities observed among human herpesviruses (Fig. 1a) suggest that these secondary structures are likely to play a role in the biology of most human herpesviruses. The presence of G-quadruplexes and their regulatory roles in eukaryotes and prokaryotes are increasingly recognized. Nonetheless, there are no systematic studies of G-quadruplexes present in large DNA viruses. This is the first systematic study of G-quadruplexes in herpesvirus genomes.

### PQS in repeat regions of herpesviruses

**Fig. 2:**
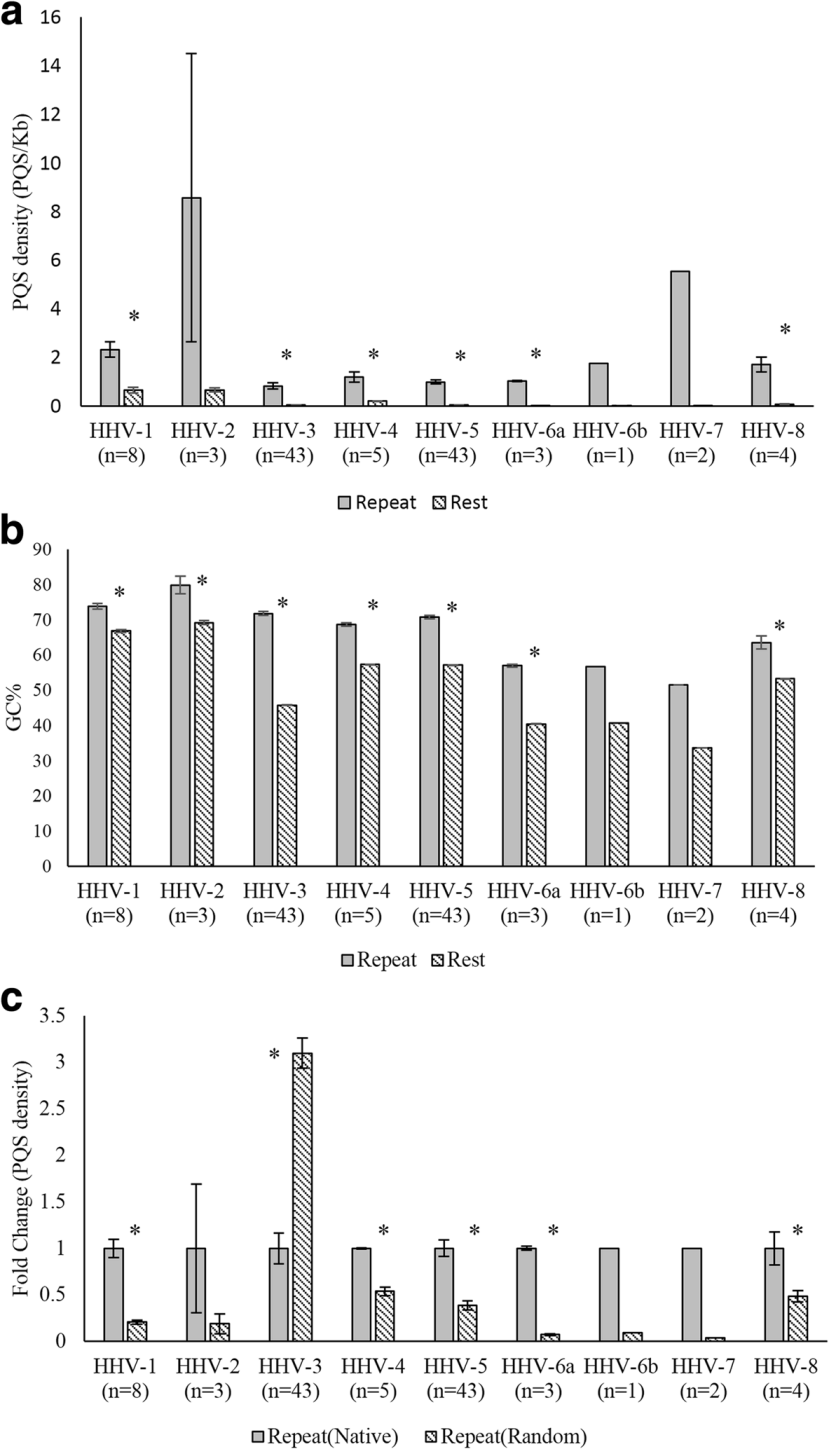
Repeat regions of herpesvirus genomes are enriched for PQS. **a** Enrichment of PQS in the repeat regions as compared to the rest of the genome: Average PQS densities in the repeat regions and in the rest of the genome of all full-length sequences available for each virus. The number of full-length sequences studied for each virus is shown in parentheses. Please see Additional file 1 for accession numbers. **b** The GC content in the repeat regions of human herpesviruses are higher than that in the rest of the genome. **c** PQS are enriched in native repeat regions within herpesvirus genomes compared to randomized repeat regions. Each repeat region in a herpesvirus genome was randomized 5 times. The fold change between average PQS density of native repeat regions and average PQS density of randomized repeat regions is plotted. HHV-3 was an exception with higher PQS densities in the randomized repeat regions. * denotes *P* < 0.01

The repeat regions in herpesvirus genomes are important for the maintenance of the episomal form of the genome; the deletion of the terminal repeats renders the virus non-infectious [26, 27]. Repeat regions have been shown to play important regulatory roles among herpesviruses [28]. Interestingly, PQS densities are significantly enriched within the repeat regions of herpesviruses as compared to that in the rest of the genome (Fig. 2a), clearly suggesting the possibility of yet unknown but important roles for G-quadruplexes in the biology of human herpesviruses. Repeat regions of herpesvirus genomes are known to be GC rich [29, 30]. Therefore we wanted to know whether higher PQS densities in the repeat region is linked to higher GC content in these regions. After analyzing the GC% of repeat regions and rest of the genome, we found that the GC% of the repeat region was significantly higher than the GC% of the rest of the genome (Fig. 2b). We therefore randomized the repeat regions to see whether high PQS densities is solely a result of high GC%. Interestingly, the native repeats regions had higher PQS densities as compared to randomized repeat regions of the respective herpesvirus genomes; HHV-3 was an exception, with higher PQS densities in the randomized repeat region sequences (Fig. 2c). Our findings confirm that the enrichment of PQS in the repeat regions of herpesviruses (with the exception of HHV-3) is neither a random event nor a function of the higher GC content within the repeat regions. To support this conclusion we performed a sliding window analysis permutation test as well. In the sliding window analysis, PQS densities in native sequences of the repeat regions (divided into 40bp sliding windows) were higher for most herpesviruses as compared to randomized sequences (Additional file 2: Figure S1b). Again, HHV-5 and HHV-3 were exceptions.

Terminal repeat regions of the human herpesviruses contain sequences essential for cleavage and packaging [31–33], indicating potential functional roles for G-quadruplexes within the repeat regions. G-quadruplexes have been identified as key players in recombination in the human genome [34]. HHV-6 and HHV-7 have telomere-like repeats at the genome termini (repeat regions) which are shown to play a role in homology mediated recombination with the human telomeric region [35].

### Enrichment of PQS in the regulatory regions of herpesvirus genome

**Fig. 3:**
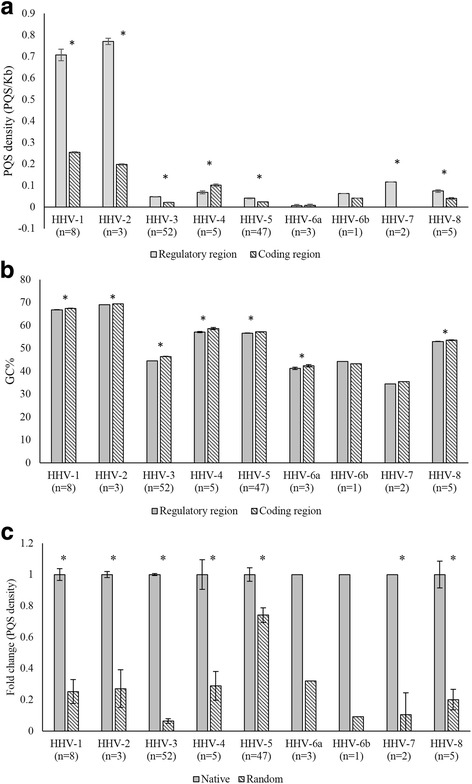
Regulatory regions of herpesvirus genomes have higher PQS densities. **a** PQS are enriched in regulatory regions as compared to coding regions: Bar graphs showing significant enrichment of PQS densities in the regulatory regions (i.e., 1000bp upstream of the start codon) as compared to that in coding regions. The number of full-length sequences studied for each virus is shown in parentheses. **b** GC content of regulatory regions is lower than that of the coding regions in herpesvirus genomes. **c** Native regulatory regions within herpesvirus genomes have significantly higher PQS densities compared to randomly generated regulatory regions within herpesvirus genomes. Each regulatory region within a herpesvirus genome was randomized 5 times. The fold change between average PQS density of native regulatory region and average PQS density of randomized regulatory regions is plotted. * denotes *P* < 0.05

Regulatory regions of the herpesvirus genome (1000 bp upstream of the start codon of a gene) are enriched for PQS densities compared to the coding regions of the genome (Fig. 3a). HHV-4 was an exception to this rule. The GC% in regulatory regions of all herpesvirus genomes were significantly lower than the GC% in the coding region; Fig. 3b. Despite their lower GC content regulatory regions had significantly higher PQS densities as compared to the coding regions. To show that the enrichment of PQS density in regulatory regions compared to coding region is not a random event we randomized the regulatory regions and calculated the PQS density in randomized regulatory regions. PQS densities in native regulatory regions (1000 bp upstream of coding regions) were significantly higher as compared to randomized regulatory regions (Fig. 3c) among hepesviruses. In addition, in the sliding window analysis, native regulatory regions of most herpesviruses (divided into sliding windows of 40 bp) showed higher PQS densities as compared to randomized sequences (Additional file 2: Figure S1c). HHV-5 and HHV-6a were exceptions, with higher PQS densities in the randomized sequences as compared to the native sequences from the regulatory regions. This finding again unambiguously indicates that the enrichment of PQS within herpesviruses is not a random event and that PQS are truly enriched in the herpesvirus promoters.

Sequences upstream of the start codon act as transcriptional and translational regulatory elements. The enrichment of PQS near the transcription start site has been used as a surrogate for putative functional roles of PQS as regulatory elements in human-, animal- and bacterial genomes [10–12]. The enrichment of PQS in the 5′ UTR has been assigned roles in translational regulation in previous reports [36, 37]. The regulatory regions in herpesviruses (1000 bp upstream of the start codon of a gene) enriched for PQS contain both the transcriptional start site and 5′ UTRs, strengthening a putative regulatory role for G-quadruplexes in human herpesviruses.

### Presence of PQS in the regulatory regions of genes common among herpesviruses

**Fig. 4:**
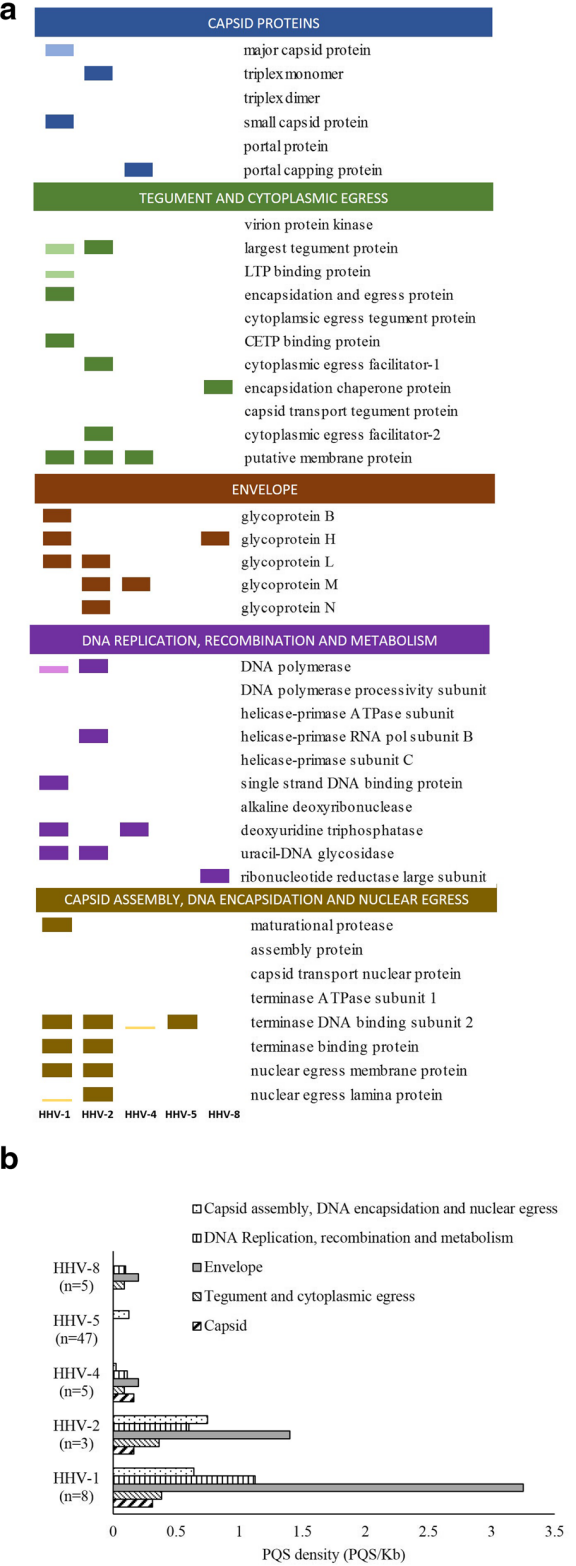
PQS are present in regulatory regions of functionally conserved genes. **a** Distribution of PQS in the regulatory region of genes which are functionally conserved across human herpesviruses. The dark colored blocks (for each colour) represent the presence of at least one PQS in the regulatory regions of genes (1000 bp upstream of start codon) among all strains (100%) of a given herpesvirus. The light colored blocks (for each colour) represent <100% conservation of at least 1 PQS in the regulatory regions of genes (1000 bp upstream of start codon) among all strains; the height of the light colored blocks indicates the proportion of strains that had at least 1 PQS conserved in the regulatory regions of a given gene. For example, if 50% of all strains have at least 1 PQS conserved in the regulatory region of a given gene, it will be depicted as a light colored block with half the height of the dark colored block (since dark colored blocks indicate that at least 1 PQS is conserved in 100% of the strains studied). HHV-6A HHV-6B and HHV-7 were not considered for this analysis as their genomes contained very few PQS outside the repeat regions. HHV-3 was excluded as there was no PQS in any of the regulatory regions of the genes that are functionally conserved across herpesviruses. The number of sequences studied for each virus are: HHV-1(*n* = 8), HHV-2 (*n* = 3), HHV-4 (*n* = 5), HHV-5 (*n* = 47), HHV-8 (*n* = 5) **b** Bar graph shows the distribution of PQS densities in the regulatory regions of the 5 functionally classes of conserved genes across human herpesviruses. The number of full-length sequences studied for each virus is shown in parentheses

The nine human herpesviruses differ significantly in their genome organization and genetic content [38]. Although human herpesviruses are evolutionary divergent, they share some core gene products or proteins which are conserved through evolution; these proteins have been classified into five major groups based on their functions: a) capsid proteins b) tegument and cytoplasmic egress c) envelope d) DNA replication, recombination and metabolism and e) capsid assembly, DNA encapsidation and nuclear egress [39] (Additional file 3: Table S2). We hypothesized that the presence of PQS within the regulatory regions of these five classes of human herpesvirus genes will further vindicate their biological role. We studied the presence of PQS in the regulatory regions of the 5 functional classes of genes conserved across human herpesviruses; a pictorial representation of whether or not at least 1 PQS is present in the regulatory region of all sequences of a given herpesvirus is shown in Fig. 4a. HHV-6a, HHV-6b and HHV-7 were excluded from the analysis as their genomes had only a few PQS outside the repeat region. In addition, HHV-3 which had no PQS in the regulatory regions of genes functionally conserved among herpesviruses was also excluded. Majority of genes functionally conserved across human herpesviruses contain one or more PQS within the regulatory regions (Fig. 4a). The average PQS densities in the regulatory region of each functional class of genes among human herpersviruses are shown in Fig. 4b. In general, high PQS densities were observed for envelope and capsid proteins.

The regulatory regions of important structural and non-structural proteins including the major capsid protein, the large tegument protein, DNA polymerase, single-stranded DNA binding protein and uracil DNA-glycosidase of several human herpesviruses had at least 1 PQS. The presence of at least 1 PQS in the regulatory regions of several genes with common functions across different herpesviruses suggest a regulatory function of G-quadruplexes in human herpesviruses.

### Unique intramolecular PQS repeats in herpesviruses

In our study we found a unique type of intramolecular PQS repeats, where a given sequence capable of forming an intramolecular G-quadruplex by itself is repeated several times within a short span in the virus genome. For example the PQS “GGGTTAGGGTTAGGGTTAGGG” in the HHV-7 genome is repeated 81 times within a 3 kb stretch of the genome and a total of 204 times in the complete genome (Table 1). We refer to these G-quadruplex repeats as repetitive G-quadruplex motifs (RGQM). Although telomere-like repeats have been reported earlier for HHV-7 [40], the presence of G-quadruplexes or intramolecular PQS repeats in HHV-7 has not been reported. Most herpesviruses contained RGQMs (Table 1). All RGQMs were confined to the repeat regions of the genome (Table 1), suggesting potentially important roles for these complex secondary structures in the biology of herpesviruses.

**Table 1:** Presence of repetitive G-quadruplex motif (RGQM) in herpesvirus genome

Virus name	Sequence	Nucleotide position	n[max]	Percentage Conservation	Genome Location
HHV-1	GGGAGGAGCGGGGGGAGGAGCGGG	106–286 & 151968–152136	7	87.5	Repeat region
	GGGAGTGGGGGTGCGTGGGAGTGGGGG	5725–5873	7	87.5	Repeat region
	GGGGCTGGGGTTGGGGTTGGGG	71612–71792	8	100	Repeat region
	GGGGAGTGGGTGGGTGGGGAGTGGG	143736–143833	4	100	Repeat region
	GGGGGCGAGGGGCGGGAGGGGGCGAGGGG^a^	25142–25448	8	87.5	Repeat region
	GGGGAGGGCTGGGGCCGGGGAGGGCTGGGG^a^	25537–25692	3	100	Repeat region
	GGGAGGAGCGGGGGGAGGAGCGGG^a^	26009–26177	8	75	Repeat region
HHV-2	GGGGCGGCTGGGGCAGGGGCGGCTGGGG	72102–72222	3	100	Repeat region
	GGGGGGACGGGGGGACGGGGGGACGGGGGG	133385–133489	5	100	Repeat region
	GGGGGTCGGGCGGGCGGGGGTCGGG	153828–154066	8	100	Repeat region
	GGGGGGCCGGGGGGACGGGGGGACGGGGGG^a^	6406–6502	4	75	Repeat region
HHV-6A	GGGTTAGGGTTAGGGTTAGGG^a^	75–411 & 151327–151645	14	100	Repeat region
HHV-6B	GGGTTAGGGTTAGGGTTAGGG^a^	77–521, 8459–8669, 153410–153842, 161780–161990	18	–	Repeat region
HHV-7	GGGTTAGGGTTAGGGTTAGGG^a^	82–222, 5938–8907, 8985–9923, 9195–9243, 9387–9651, 143128–143262, 148984–151965, 152031–152169, 152253–152349, 152445–152715	82	100	Repeat region
HHV-8	GGGATGGGGGTGTGGGATGGGGG	29928–30032	5	80	Repeat region

n[max] is the maximum number of iterations (n[max]) of a given RGQM

The percent conservation of each RGQM indicated in the tables is based on the presence of a minimum of three iterations of a given RGQM in all available sequences [i.e., HHV-1 (*n* = 8), HHV-2 (*n* = 3), HHV-3 (*n* = 52), HHV-4 (*n* = 5), HHV-5 (*n* = 47), HHV-6A (*n* = 3), HHV-6B (*n* = 1), HHV-7 (*n* = 2), HHV-8 (*n* = 5) for a given virus.]

^a^RGQM present in the reverse complement of the strand represented in NCBI

We looked for conservation of RGQMs in all available full-length sequences of human herpesviruses (*n* = 126). We defined conservation of RGQM as the presence of three or more identical PQS in a nucleotide stretch of defined length. For example in a given herpesvirus if 7 out of 9 sequences have three or more identical PQS in a nucleotide stretch of defined length, the percentage conservation is estimated as 77% (i.e., 7 out of 9). Interestingly, for a given human herpesvirus, majority of the RGQMs were at least 80% conserved among the sequences analyzed (Table 1).

Stretches of G-rich sequences that can form intramolecular G-quadruplex structures are reported in human telomeres [2]. Recently, Artusi et al., have reported the presence of G-quadruplex repeats in the HHV-1 genome [17]. It is well known that G-quadruplex structures are very stable. G-quadruplex superstructures known as G-wires formed by the self-assembly of repetitive G-quadruplexes are more stable than a single G-quadruplex [41]. Therefore it is likely that the RGQMs are much more stable than an individual G-quadruplex structure, suggesting potentially interesting functions for RGQMs among herpesviruses.

### Immediate early genes of alphaherpesviruses are enriched for PQS

The expression of genes in herpesviruses occurs in a sequential and coordinated manner; this results in three groups of temporally expressed proteins referred to as the immediate early, early and late proteins. The immediate early genes are the first set of genes to be expressed followed by the early genes and then the late genes. The factors responsible for the temporal control of viral gene expression are poorly understood [42].

**Fig. 5:**
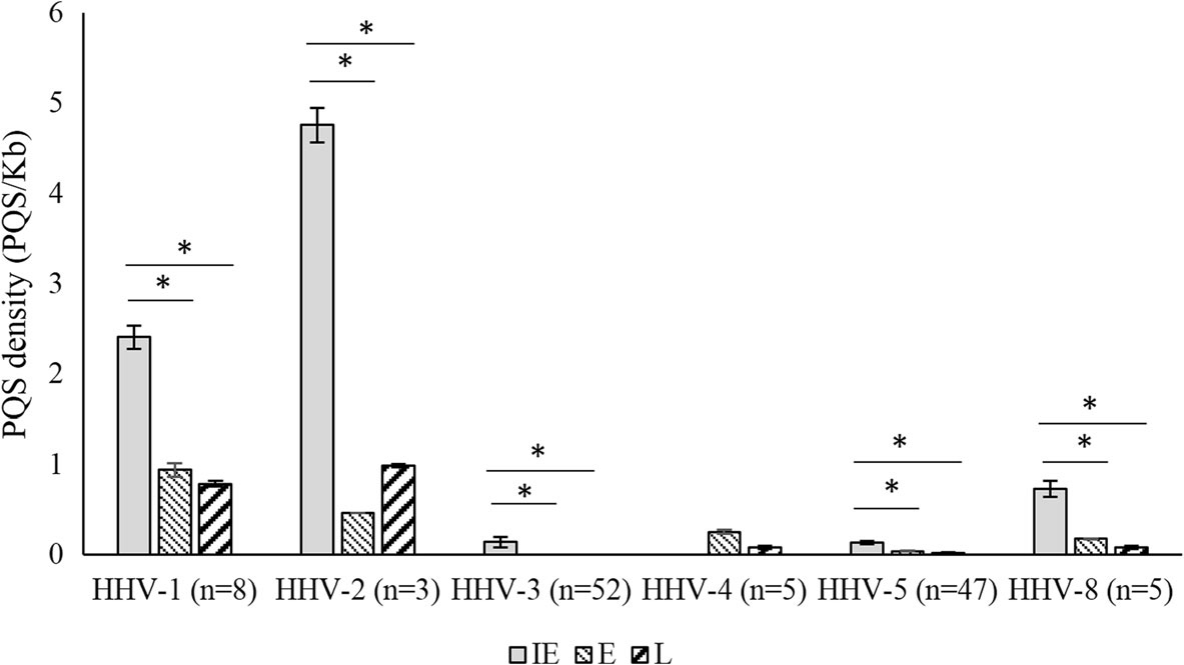
Immediate early genes have higher PQS densities compared to early and late genes. Bar graph showing average PQS densities in the regulatory regions (1000bp upstream of the start codon) of immediate early (IE) genes as compared to early (E) genes and late (L) genes. The number of full-length sequences studied for each virus is shown in parentheses. Among herpesviruses (HHV-1, HHV-2, HHV-3, HHV-5, HHV-8) the regulatory regions of IE genes had several fold higher PQS densities as compared to that of E genes or L genes (*P < 0.05)

We analyzed the distribution of PQS in the regulatory regions of temporally regulated herpesvirus genes (Please see Additional file 4: Table S3 for list of temporally regulated genes analyzed). HHV-6 and HHV-7 were excluded from this analysis due to the near absence of PQS outside the terminal repeats (Fig. 1b). Interestingly, among most herpesviruses (HHV-1, HHV-2, HHV-3, HHV-5 and HHV-8) the regulatory regions of immediate early genes had significantly higher PQS densities as compared to that of early genes and late genes (*P* < 0.05, Fig. 5); this trend was not obvious for HHV-4. HHV-4 had the lowest number of genes characterized as immediate early genes (Additional file 4: Table S3). The presence of very few immediate early genes in HHV-4 precludes meaningful interpretation of results among temporally regulated genes for this virus.

Immediate early genes are the first set of herpesvirus genes to be activated and transcribed. Thus the regulation of immediate early genes is largely dependent on host transcription regulatory factors and elements rather than on viral factors [20]. Considering the well-established role of G-quadruplexes as regulators of transcription in the human genome, high PQS densities and the presence of PQS within the regulatory regions of a large proportion of immediate early genes in herpesviruses may potentially represent virus mimicry of host regulatory elements. Our findings support a potential regulatory role for PQS in the regulation of immediate early genes encoded by alphaherpesviruses (HHV-1, HHV-2 and HHV-3); this is particularly interesting as alphaherpesviruses replicate much faster (short reproductive cycle) than beta- and gamma-herpesviruses [43]. Based on our results, G-quadruplex destabilizing agents may merit testing as potential inhibitors of alphaherpesviruses.

### Experimental evidence of G-quadruplex formation

CD spectroscopy is the most widely used method for studying the formation of G-quadruplexes. CD spectroscopy analysis allows distinction between parallel and anti-parallel G-quadruplex structures. A positive peak near 260nm and a negative peak near 240nm indicates the formation of parallel G-quadruplexes [2]. A positive peak around 290nm and a negative peak around 260nm is suggestive of anti-parallel G- quadruplexes. A hybrid type G-quadruplex would show two positive peaks, one at 290nm and the other at 260nm [44] along with a negative peak at 240nm.

We used CD spectroscopy to ascertain if the PQS (as predicted by quadparser) truly form G-quadruplexes. The CD profiles of all the 15 randomly selected PQS oligonucleotides studied (Additional file 5: Table S4) confirmed the formation of either parallel or hybrid G-quadruplex structures (Additional file 6: Figure S2). No anti-parallel structures were observed. The formation of G-quadruplexes by all the 15 (100%) randomly selected PQS oligonucleotide sequences suggests that most if not all of the PQS reported in this study are likely to form quadruplex structures as oligonucleotides.

### G-quadruplexes regulate gene expression in human herpesviruses

Having analyzed the genomic distribution and the enrichment of PQS at specific genomic locations within human herpesvirus genomes we chose 3 genes, namely: UL2 (from HHV-1), UL24 (from HHV-1) and K15 (from HHV-8) for functional analysis. The selection of these 3 genes was based on (a) Availability of genomic DNA. HHV-1 and HHV-8 DNA were available to us (b) The presence of at least one PQS in the regulatory region of the gene (c) Conserved functions across all human herpesviruses or genes with a well-established role in viral pathogenesis. The UL2 gene codes for uracil DNA-glycosidase [45], A DNA repair protein and the UL24 gene codes for a nucleolin transporter protein [46] ;essential for efficient viral replication. These two proteins are present in all human herpesviruses. The K15 gene is an elicitor of signal transduction pathways [47] and has homologues in other human herpesviruses [48]. There were other genes matching the above-mentioned criteria; UL2 (HHV-1), UL24 (HHV-1) and K15 (HHV-8) were randomly selected for functional studies from the list of genes meeting the above criteria.

#### The PQS oligonucleotides in UL2, UL24 and K15 promoters form G-quadruplexes

**Fig. 6:**
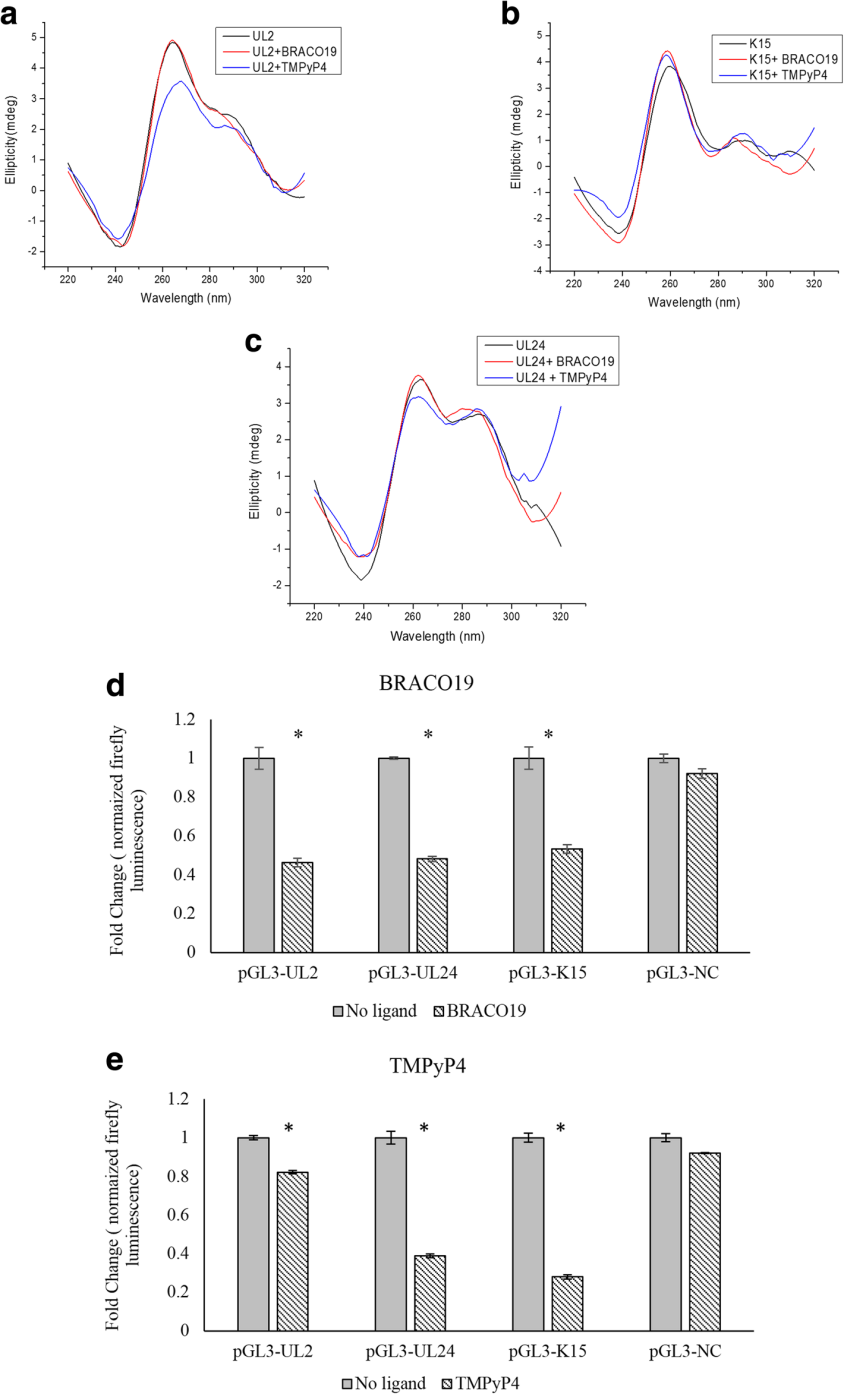
PQS present in the promoter region of UL2, UL24 and K15 gene forms G-quadruplex and regulates promoter activity. CD spectroscopy showing formation of G-quadruplex structures in oligonucleotides from the promoters of **a** UL2, **b** UL24 and **c** K15 genes in the presence ad absence of G-quadruplex binding ligands TMPyP4 (10μM) and BRACO19 (10μM); these genes were selected for functional studies. **d** Promoter activity of G-quadruplex containing promoter regions of pGL3-UL2, pGL3-UL24 and pGL3-K15 with and without BRACO19 as measured with a dual luciferase assay. **e** Promoter activity of G-quadruplex containing promoter regions of pGL3-UL2, pGL3-UL24 and pGL3-K15 with and without TMPyP4 as measured with a dual luciferase assay. The data presented in **d** and **e** show firefly luciferase levels (promoter activity) normalized with Renilla luciferase levels (internal control). A negative control, pGL3-NC (expression constructs with HHV-8 ORF50 promoter that does not contain G-quadruplexes) was also tested similarly. The addition of BRACO19 (10μM) and TMPyP4 (50μM) was linked to significant reduction in promoter activity of pGL3-UL2, pGL3-UL24 and pGL3-K15; but the gene expression from the negative control (pGL3-NC) was not influenced by the addition BRACO19 and TMPyP4. Experiments were done in in triplicate and average values are plotted. *denotes *P* < 0.005

CD spectroscopy of PQS oligonucleotides of UL2, UL24 and K15 (Additional file 7: Table S5) confirmed the formation of a hybrid type of G-quadruplex (demonstrated by a sharp positive peak near 260nm, a shoulder at 290nm and a negative peak near 240nm) in vitro (Fig. 6a, b and c). To confirm formation of G-quadruplex structures in these 3 oligos, different salt and buffer combinations were used. K+ ions induces formation of parallel structures whereas Na + induces formation of anti-parallel structures [2]. CD spectra of PQS oligonucleotides in the same buffer containing Na + demonstrated formation of antiparallel or hybrid structures having a sharp positive peak at 290nm. The change in spectral behavior in the presence of different monovalent cations [49] also proves formation of G-quadruplex structures (Additional file 8: Figure S3a). Formation of G-quadruplex structure were also observed in Tris EDTA buffer (10mM Tris, 1mM EDTA, pH: 8) containing KCl (100mM) (Additional file 8: Figure S3b). All three oligonucleotides (UL2, UL24 and K15) retained their hybrid G-quadruplex structures in sodium cacodylate buffer and KCl (100mM) when G-quadruplex binding ligands TMPyP4 or BRACO19, were added (Fig. 6a, b and c).

#### BRACO19 and TMPyP4 stabilizes G quadruplexes formed in PQS oligonucleotides of UL2, UL24 and K15

TMPyP4 and BRACO19 are known to bind to G-quadruplexes and stabilize the G-quadruplex structures [3, 50, 51]. Our CD melting studies reveal a stabilizing effect of both the ligands on the UL2, UL24 and K15 G-quadruplexes as evidenced by the increase in Tm (melting temperature) after the addition of the ligand (Table 2).

**Table 2:** Melting temperature of PQS oligonucleotides in the absence and presence of TMPyP4 or BRACO19

PQS OLIGONUCLEOTIDES	ΔTM^a^(°C) WITH TMPYP4	ΔTM^a^(°C) WITH BRACO19
UL2	20	8
UL24	13	6
K15	6	8

^a^ΔTm = Tm(oligo + ligand)-Tm(oligo)

#### G-quadruplexes regulate UL2, UL24 and K15 gene expression

After demonstrating the formation of G-quadruplexes in the oligonucleotides from promoters of UL2, UL24 and K15 and their stabilization in the presence of TMPyP4 or BRACO19, we analyzed the influence of the ligands on gene expression using luciferase reporter constructs driven by G-quadruplex-containing promoters of UL2 (pGL3-UL2), UL24 (pGL3-UL24) and K15 (pGL3-K15) (please see Methods section for details). The luciferase expression of pGL3-NC (luciferase expression is driven by promoter of ORF-50 that does not contain any G-quadruplexes) was comparable in untreated HeLa cells (no ligand added) and HeLa cells with 10μM of BRACO19 or 50μM of TMPyP4, Fig. 6d and e; this finding confirms that BRACO19 and TMPyP4 does not alter gene expression from promoters lacking G-quadruplex structures. Interestingly, the addition of BRACO19 and TMPyP4 to cells transfected with pGL3-UL2, pGL3-UL24 and pGL3-K15 led to a significant reduction in luciferase expression in all three constructs (*P* < 0.005; Fig. 6d and e). Stabilization of the UL24, UL2 and K15 G-quadruplexes by BRACO19 and TMPyP4 and the inhibition of gene expression following addition of BRACO19 and TMPyP4 suggest that G-quadruplexes in the promoter region of these three genes are negative regulators of gene expression.

G-quadruplexes in the promoter regions of the human genome have been reported as inhibitors of gene expression [3, 22]. Our findings on the G-quadruplex containing promoters of UL2, UL24 and K15 provide the first evidence of G-quadruplexes as regulators of herpesvirus gene expression. To the best of our knowledge, our findings provide the first experimental evidence demonstrating a regulatory role for G-quadruplexes within promoters of DNA viruses.

We believe that our findings that report PQS within regulatory regions are regulators of human herpesviruses gene expression is particularly important as regulation of gene expression among this group of pathogens remains poorly understood and is further complicated by (a) temporally regulated gene expression patterns (b) major changes in gene expression profiles in latency and reactivation (c) regulation of gene expression by both virus- and host-related factors.

## Conclusion

Herpesviruses are ubiquitous human pathogens and they often mimic regulatory elements of the host. In this systematic study of DNA G-quadruplexes in human herpesviruses we report several interesting findings on the presence and distribution of PQS at specific genomic locations including (a) The high PQS densities reported in our study for human herpesviruses are the highest reported for any genome studied in literature (b) Significant enrichment of PQS in the repeat regions and in the regulatory regions of human herpesviruses, suggesting a potential regulatory role for G-quadruplexes (c) The presence of PQS in the regulatory regions of the functionally conserved genes present across human herpesviruses (d) A potential role for PQS in the regulation of immediate early genes among most herpesviruses.

We report the presence of repetitive G-quadruplex motifs (RGQM), which are unique intramolecular G-quadruplex repeats, across human herpesvirus genomes. We experimentally confirm the formation of G-quadruplexes in a selected subset of PQS using CD spectroscopy. Functional studies on 3 PQS-containing promoters of herpesviruses using reporter assays suggest a role for G-quadruplexes in modulating gene expression in herpesviruses.

In sum, the abundance of PQS within human herpesviruses, their enrichment at specific genomic locations in human herpesviruses and their preferential enrichment in the regulatory regions of immediate early genes compared to early and late genes indicates functional roles for PQS in the biology of human herpesviruses. We believe that our findings provide the essential framework for a plethora of studies on the role of G-quadruplexes in the biology of herpesviruses.

## Methods

### Virus sequences

Accession numbers for full-length herpesvirus genome sequences were obtained from the ViPr (http://www.viprbrc.org) database [52]. The full-length sequences were retrieved from NCBI GenBank. A total of 126 sequences were analyzed; this includes all available full length sequences of human herpesviruses (as on December 20, 2014) whose coding DNA sequences were annotated in Vipr. All the transgenic strains were eliminated from the analysis. The accession numbers are listed in Additional file 1. The nucleotide positions and repeat regions in the human herpesviruses were mapped using information available at NCBI Genbank (http://www.ncbi.nlm.nih.gov).

### PQS mapping

- Analysis of PQS in the full-length genome
  Quadparser, a C program developed by Balasubramaniun and group was used to map PQS in herpesvirus genomes. Default parameters, 1) Minimum G-tetrad =3 and 2) Loop length = 1 to 7 were used [53]. Since all viruses analyzed are double-stranded in nature, the presence of PQS was analyzed in both the strands of DNA. Overlapping PQS identified by quadparser were excluded to avoid inappropriate and falsely elevated PQS numbers.
- Analysis of PQS in repeat regions:
  Herpesvirus genomes contain direct and inverted repeats at the termini and within the genome. The repeat regions of herpesviruses, as annotated in Genbank were analyzed for PQS densities. Herpesvirus sequences for which repeat regions in the genome were not annotated in Genbank were excluded from the analysis. The repeat regions of a total of 112 full-length sequences were analyzed.
- Analysis of PQS in putative regulatory regions and coding regions of herpesvirus genome:
  Coding DNA sequences were analyzed for PQS densities. The CDS was extracted from FeatureExtract 1.2 server database [54] for each sequence analyzed. Majority of promoters/regulatory regions of herpesvirus encoded genes are not mapped. A previous study investigating herpesvirus regulatory regions made significant discoveries based on the assumption that the 1000bp upstream region of herpesvirus genes contained putative regulatory regions [55]. Similarly, we assumed that putative regulatory regions for human herpesviruses lie within 1000bp upstream of herpesvirus encoded genes; 1000bp upstream sequences for all genes were also retrieved from FeatureExtract 1.2 server database. PQS densities were calculated for the putative regulatory regions and then compared to that within coding regions.
- Randomization of sequences:
  Using an in-house program each of the 126 herpesvirus whole genome sequences were randomized 5 times [6] (this was done without changing the overall nucleotide composition). All the regulatory and repeat regions of each of the 126 herpesvirus sequences were also randomized 5 times. PQS were mapped in the randomized sequences generated and were compared to that in the native sequences. In addition, we also randomized (random shuffling of sequences without changing the overall mononucleotide composition) sequences using a sliding window size of 40bp that is slid along the length of the sequence (whole genome, regulatory regions and repeat regions) that is analyzed. For example, if the whole genome sequence is 150,000bp it will be analyzed in 149,961 windows of 40bp each (i.e., 1-40bp, 2-41bp, 3-42bp etc.) and for each window the sequences will be randomized 5 times and analyzed for PQS. Then the PQS densities in the randomized sequences were compared with that in the respective native sequences.

### Mapping PQS in the regulatory regions of functionally conserved genes in human herpesviruses

Distinct functional classes of genes have been reported to be conserved across human herpesviruses including genes encoding a) capsid proteins b) tegument proteins and proteins involved in cytoplasmic egress c) envelope proteins d) proteins involved in DNA replication, recombination and metabolism e) proteins pertaining to capsid assembly, DNA encapsidation and nuclear egress [39]. The list of functionally conserved genes among human herpesviruses is provided in Additional file 3: Table S2. We analyzed the presence of PQS within putative regulatory regions (1000bp upstream of the start codon) of distinct functional classes of genes that are conserved across human herpesviruses.

### Mapping of PQS in temporally regulated genes of herpesviruses

Many herpesvirus-encoded genes are classified as belonging to one of the three classes (i.e., immediate early or early or late) of temporally regulated genes; nonetheless, a comprehensive categorized list of genes is not readily available for most herpesviruses. We searched the published literature extensively [24, 56–63] and compiled a list of immediate early, early, and late genes for each human herpesvirus (Additional file 4: Table S3). We then used this list to analyze the presence of PQS within the putative regulatory region (i.e., 1000bp upstream of the start codon) of temporally regulated genes. The putative regulatory sequences (1000bp upstream of CDS) were retrieved from FeatureExtract 1.2 server database for all temporally regulated genes listed in Additional file 3: Table S2; this was done for all sequences studied. HHV-6a, HHV-6b and HHV-7 sequences were excluded from this analysis as they contained very few PQS (≤10) outside the repeat regions.

### CD spectroscopy

A total of 15 viral PQS were randomly selected using the “randbetween” function in MS excel to analyze their ability to form G-quadruplex using CD spectroscopy; Additional file 5: Table S4. All oligonucleotides were purchased from Integrated DNA technologies (IDT).

A J 815 spectrophotometer (JascoInc, Japan) was used to conduct CD experiments. Oligonucleotides at 10μM concentration prepared in sodium cacodylate buffer (10mM) containing 100mM KCl were used for CD experiments. The oligonucleotides were heated at 95 °C for 5 mins and cooled slowly to room temperature [44]. A quartz cuvette having a pathlength of 1mm was used. CD scans were taken at 20 °C over a range of 220-320nm. An average of 3 scans was taken with a bandwidth of 0.5nm, step size of 1nm and a time per point of 1s to plot the final trace. A sample containing only sodium cacodylate buffer and KCl, treated in the same manner was used as the blank.

CD spectroscopy was also performed on oligonucleotides of PQS predicted in the promoter region of UL24, UL2 and K15 (Additional file 7: Table S5) in the presence and absence of widely used G-quadruplex ligands TMPyP4 and BRACO19. The UL24, UL2 and K15 PQS were allowed to form G-quadruplexes, TMPyP4 and BRACO19 were added at a concentration of 10μM each and incubated in the dark for 10mins prior to spectral measurements. In addition, CD spectra were also analyzed with a) sodium cacodylate buffer containing NaCl (100mM) and b) tris EDTA buffer (1×) containing KCl (100mM) for UL2, UL24 and K15 PQS oligonucleotides.

CD melting was performed on UL24, UL2 and K15 PQS oligonucleotides in the presence and absence of G-quadruplex binding ligands TMPyP4 and BRACO19. The ellipticity change was monitored with temperature at a fixed wavelength of 262nm. Denaturation was monitored at a rate of 0.5 °C/min. Tm was calculated using first derivative method.

### Cloning of promoter regions

Three genes that had a PQS within the promoter regions, namely UL2, UL24 and K15 were selected for functional studies. In order to use a virus-derived promoter without a G-quadruplex as the negative control we amplified the promoter region of ORF 50 from HHV-8 that does not have a PQS within its promoter region. The PQS-containing promoter regions of the UL2 and UL24 genes were amplified from HHV-1 DNA (courtesy Prof. Asha Mary Jesudason and Prof. Rajesh Kannangai, CMC&H, Vellore, India) and the PQS containing promoter region of K15 and non-PQS containing promoter region of ORF 50 were amplified from HHV-8 DNA (courtesy Dr.Tathagata Choudhuri, Visva Bharati University, Bolpur, India). The amplified products were digested with respective restriction enzymes (indicated in italics in Additional file 9: Table S6) and cloned into a promoter less firefly luciferase vector (pGL3 basic vector); the luciferase expression from this vector is dependent solely on the putative regulatory element cloned. The primers used for amplification and cloning are given in Additional file 9: Table S6. The plasmids containing promoter regions of UL2, UL24, K15 and ORF 50 (negative control) with a luciferase reporter are subsequently referred to as pGL3-UL2, pGL3-UL24, pGL3-K15, pGL3-NC respectively.

### Luciferase assays

HeLa cells were seeded in a 24-well plate at a concentration of 3×10^4^ cells and co-transfected with pGL3-UL2 or pGL3-UL24 or pGL3-K15 along with pRL-TK (a renilla luciferase reporter construct with a thymidine kinase promoter) at a ratio of 25:1 (500ng for pGL3 constructs and 20ng for pRL-TK) using lipofectamine 2000 (Invitrogen) according to manufacturer’s protocol. pRL-TK serves as an internal control for normalization. TMPyP4 or BRACO19 were added 2 h after transfection at a concentration of 50μM and 10μM respectively to avoid interference, if any, with transfection. Cells were harvested after 24 h of transfection. Firefly and renilla luminescence were recorded using a luminometer (Berthold, USA) and the assay was performed using a dual luciferase reporter assay system (Promega) according to the manufacturer’s protocol.

### Statistical analysis

Student’s *t*-test was used to determine statistical significance. Box plots and bar graphs were plotted using MS excel. *P* values of < 0.05 were considered significant. In the box plots the box represents 1^st^ to 3^rd^ quartile. The central horizontal line represents the median value, and the positive and negative bars represent the maximum and the minimum values unless otherwise stated. Mean ± SE was used for all the bar graphs plotted unless otherwise state.

## Additional files

Additional file 1: Table S1. Accession numbers. Accession numbers of human herpesviruses sequences analyzed. (PDF 86 kb)
Additional file 2: Figure S1. Randomization using sliding window analysis. **a** Bar graph shows enrichment of PQS in the native full length genome sequences of most herpesviruses as compared to randomized sequences using the sliding window approach. Native sequences were divided into 40bp sliding windows and thereafter each sequence was randomized 5 times. **b** Bar graph shows comparison between native and randomized sequences of repeat regions using the sliding window approach. PQS densities are higher in native repeat region sequences of most herpesviruses compared to randomized repeat region sequences. **c** Bar graph comparing native and randomized sequences of regulatory regions of herpesviruses using the sliding window approach. PQS densities are higher in native regulatory region sequences of most herpesviruses compared to randomized regulatory region sequences. *denotes *P* < 0.05 (PDF 207 kb)
Additional file 3: Table S2. Genes conserved among human herpesviruses. List of genes that are functionally conserved across human herpesviruses. (PDF 148 kb)
Additional file 4: Table S3. Temporally regulated genes. List of herpesvirus genes categorized as immediate early, early and late genes. (PDF 89 kb)
Additional file 5: Table S4. PQS oligonucleotides. Names and sequences of virus PQS oligonucleotides randomly selected for CD spectroscopy. (PDF 92 kb)
Additional file 6: Figure S2. CD spectroscopy. CD spectroscopy profiles of 15 randomly selected deoxyoligonucleotides from herpesvirus genome as predicted by quadparser. C-myc is used as the positive control for a parallel G-quadruplex. An oligonucleotide that is reported to form a hybrid G-quadruplex [49] is used as a hybrid PQS control. A G-rich sequence that could not form a G-quadruplex is included as a negative control [G-rich (-) control].A positive peak near 260nm is indicative of parallel (P) G-quadruplexes; a positive peak near 290nm is suggestive of an antiparallel (AP) G- quadruplex. A positive peak at both 260nm and 290nm is indicative of a hybrid (H) G-quadruplex. (PDF 207 kb)
Additional file 7: Table S5. Promoter PQS oligonucleotides. Sequence of PQS oligonucleotides from the promoter region of UL24, UL2, and K15. (PDF 82 kb)
Additional file 8: Figure S3 CD spectroscopy. CD spectroscopy of UL2, Ul24 and K15 PQS oligonucleotides using **a** Sodium cacodylate buffer and NaCl **b** Tris EDTA buffer and KCl. (PDF 178 kb)
Additional file 9: Primer sequences. Primer sequences used in this study. (PDF 149 kb)

## Abbreviations

CD: Circular dichroism
HHV: Human herpesvirus
PQS: Putative quadruplex sequences
