# Supplementary material for: Genome-wide analysis of G-quadruplexes in herpesvirus genomes
Source: BMC Genomics. 2016 Nov 21;17:949. doi: 10.1186/s12864-016-3282-1 (PMC5117502; doi:10.1186/s12864-016-3282-1)
Supplement: Additional file 1: Table S1. — Accession numbers. Accession numbers of human herpesviruses sequences analyzed. (PDF 86 kb) [file 12864_2016_3282_MOESM1_ESM.pdf]

Table S1. Accession numbers of human herpesviruses sequences analyzed.

Virus name	Strains accession number
Human herpesvirus 1	*JN555585, NC_001806 , X14112, GU734771, GU734772, JQ673480, JQ780693, KF498959*
Human herpesvirus 2	*JN561323, NC_001798 , Z86099*
Human herpesvirus 3	*DQ479957, JN704697, JN704698, DQ479955, DQ479956, DQ479961, DQ479962, DQ479963, DQ479958, DQ479959, DQ479960, KC847290, AY548171, DQ457052*, NC_001348 , JQ972913, DQ452050*, AJ871403, DQ479954, JQ972914, JF306641*, AY548170, DQ674250*, DQ479953, EU154348*, KC112914*, DQ008354, DQ008355,, JN704696, JN704700, JN704707, JN704690, JN704705, JN704704, JN704708, JN704702, JN704701, JN704710, JN704695, JN704692, JN704691, JN704709, JN704699, JN704703, JN704706, JN704693, JN704694, KF811485, KJ767491*, KJ767492*, KJ808816*, X04370*
Human herpesvirus 4	*DQ279927, AJ507799, NC_007605 , V01555, AY961628*
Human herpesvirus 5	*GQ221974, GQ466044, JX512197, BK000394, FJ527563, X17403, GU179291, KC519320, KC519321, KC519322, KC519323, KC519319, JX512198, KJ426589, JX512199, JX512203, GQ221973, JX512204, JX512205, JX512200, GQ396663, JX512206, JX512207, JX512201, JX512208, GQ396662, JX512202, HQ380895*, GQ221975, AY446894, KM192298, KM192299, KM192300, KM192301, KM192302, NC_006273 , EF999921*, KF297339, GU937742, AC146905*, AY315197*, FJ616285, KF021605, GU179290, GU179288, KJ361971, GU179289*
Human herpesvirus 6a	*KC465951, KJ123690, NC_001664*
Human herpesvirus 6b	*NC_000898*
Human herpesvirus 7	*AF037218, NC_001716*
Human herpesvirus 8	*JQ619843*, AF148805, NC_009333 , GQ994935, HQ404500*

* Sequences without annotated repeat sequences; excluded for analysis for Fig 2.
