# Supplementary figures and images for: Genome-wide analysis of G-quadruplexes in herpesvirus genomes
Source: BMC Genomics. 2016 Nov 21;17:949. doi: 10.1186/s12864-016-3282-1 (PMC5117502; doi:10.1186/s12864-016-3282-1)

**Figure S1**

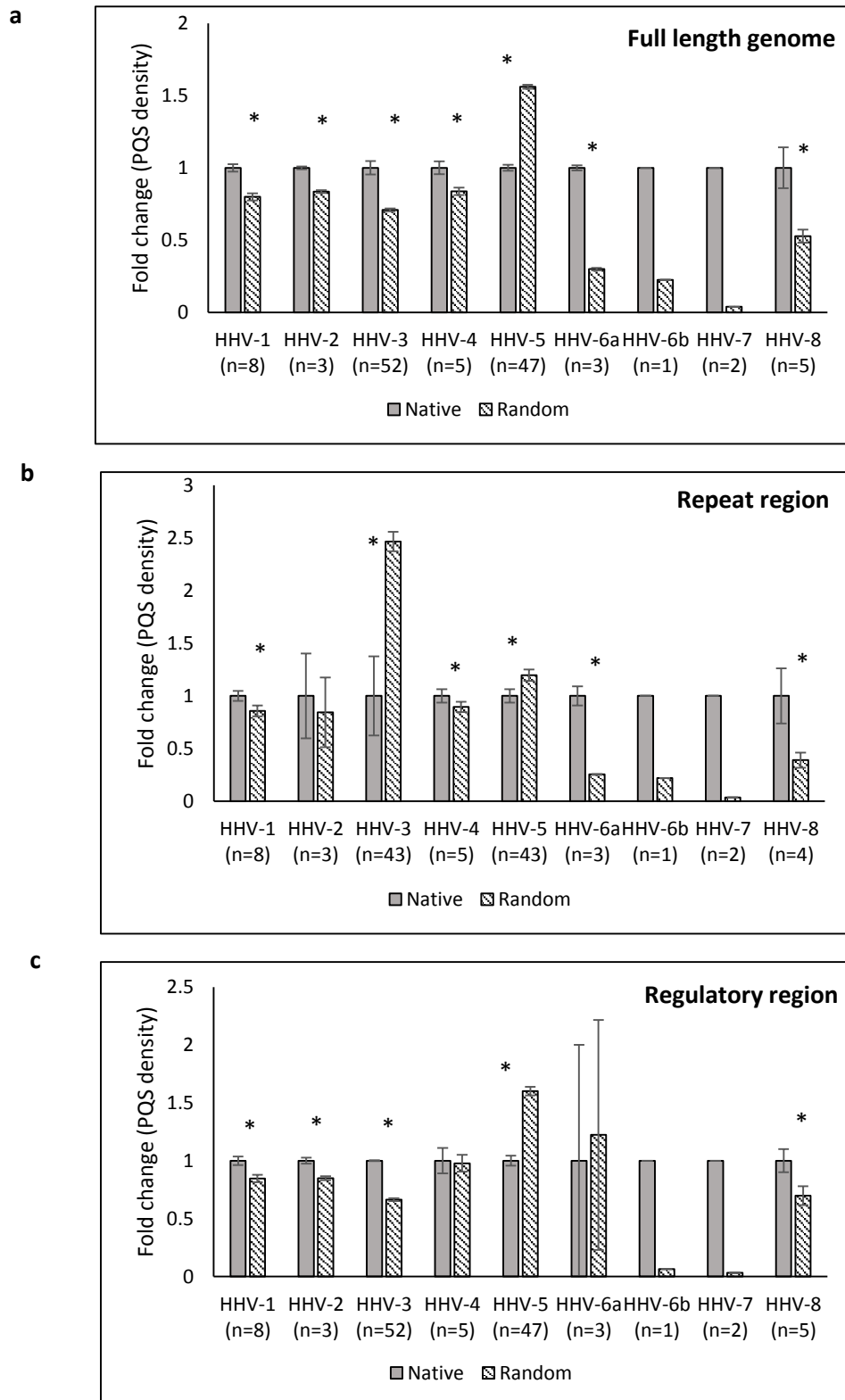

Supplement: Additional file 2: Figure S1. — Randomization using sliding window analysis. a Bar graph shows enrichment of PQS in the native full length genome sequences of most herpesviruses as compared to randomized sequences using the sliding window approach. Native sequences were divided into 40bp sliding windows and thereafter each sequence was randomized 5 times. b Bar graph shows comparison between native and randomized sequences of repeat regions using the sliding window approach. PQS densities are higher in native repeat region sequences of most herpesviruses compared to randomized repeat region sequences. c Bar graph comparing native and randomized sequences of regulatory regions of herpesviruses using the sliding window approach. PQS densities are higher in native regulatory region sequences of most herpesviruses compared to randomized regulatory region sequences. *denotes P < 0.05 (PDF 207 kb) [file 12864_2016_3282_MOESM2_ESM.pdf]

Figure S2

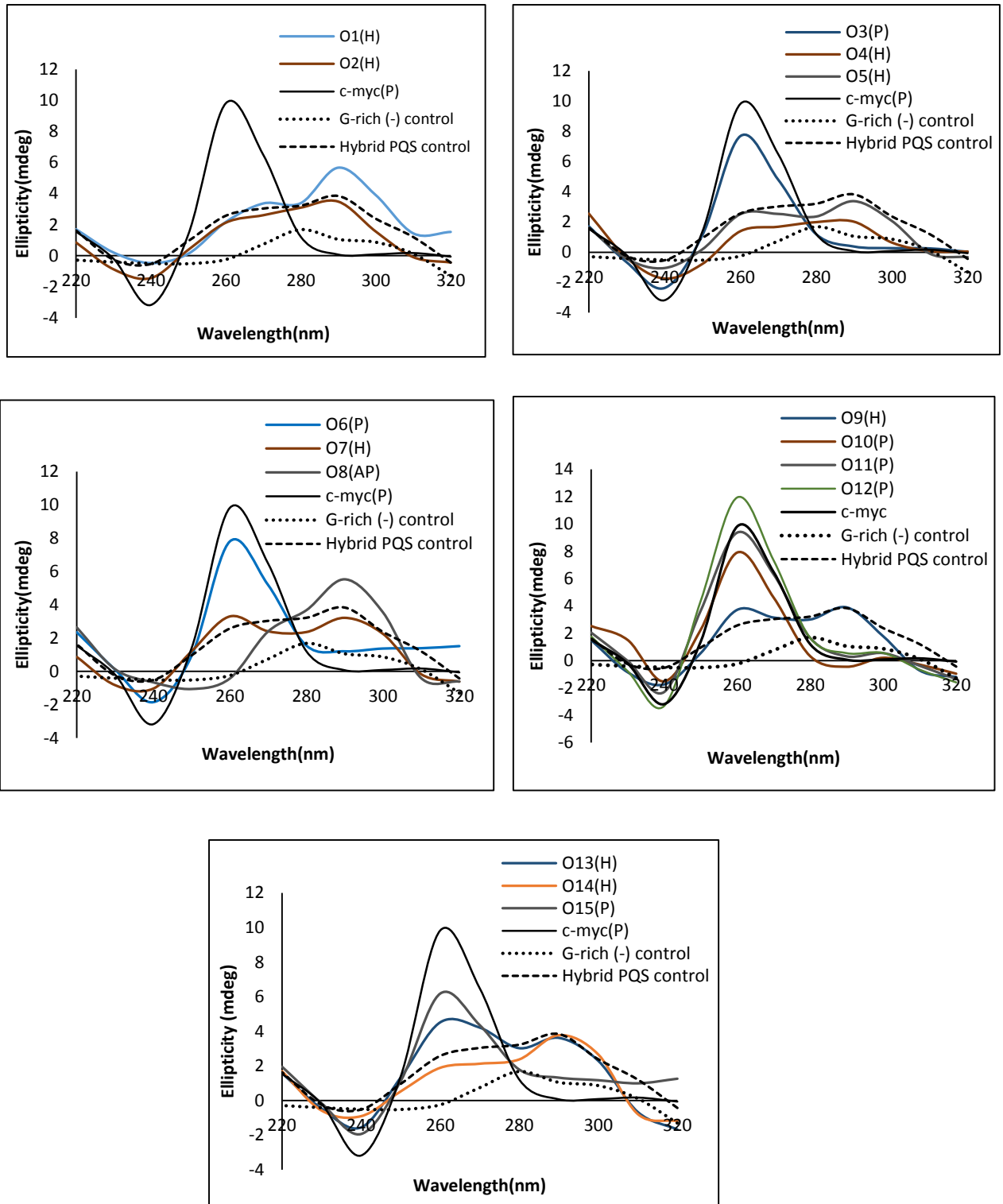

Supplement: Additional file 6: Figure S2. — CD spectroscopy. CD spectroscopy profiles of 15 randomly selected deoxyoligonucleotides from herpesvirus genome as predicted by quadparser. C-myc is used as the positive control for a parallel G-quadruplex. An oligonucleotide that is reported to form a hybrid G-quadruplex [49] is used as a hybrid PQS control. A G-rich sequence that could not form a G-quadruplex is included as a negative control [G-rich (-) control].A positive peak near 260nm is indicative of parallel (P) G-quadruplexes; a positive peak near 290nm is suggestive of an antiparallel (AP) G- quadruplex. A positive peak at both 260nm and 290nm is indicative of a hybrid (H) G-quadruplex. (PDF 207 kb) [file 12864_2016_3282_MOESM6_ESM.pdf]

**Figure S3**

**a**

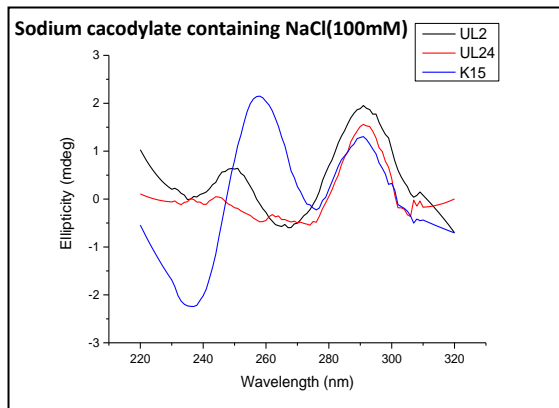

**b**

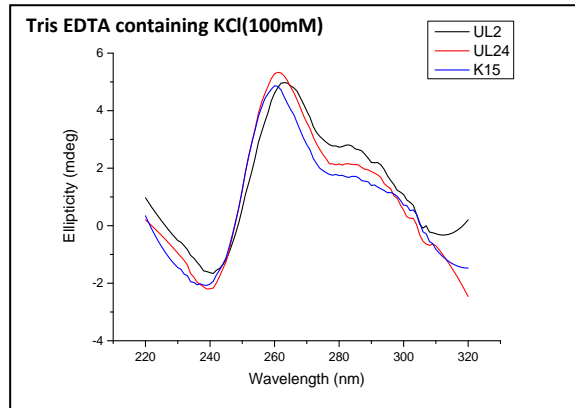

Supplement: Additional file 8: Figure S3 — CD spectroscopy. CD spectroscopy of UL2, Ul24 and K15 PQS oligonucleotides using a Sodium cacodylate buffer and NaCl b Tris EDTA buffer and KCl. (PDF 178 kb) [file 12864_2016_3282_MOESM8_ESM.pdf]
