# Supplementary material for: Genome-wide analysis of G-quadruplexes in herpesvirus genomes
Source: BMC Genomics. 2016 Nov 21;17:949. doi: 10.1186/s12864-016-3282-1 (PMC5117502; doi:10.1186/s12864-016-3282-1)
Supplement: Additional file 3: Table S2. — Genes conserved among human herpesviruses. List of genes that are functionally conserved across human herpesviruses. (PDF 148 kb) [file 12864_2016_3282_MOESM3_ESM.pdf]

Table S2. Genes that are functionally conserved across human herpesviruses[1].

Common name	HHV-1/2	HHV-3	HHV-5	HHV-6/7	HHV-4	HHV-8
Capsid						
major capsid protein	UL19	40	UL86	U57	BcLF1	ORF25
triplex monomer	UL38	20	UL46	U29	BORF1	ORF62
triplex dimer	UL18	41	UL85	U56	BDLF1	ORF26
small capsid protein	UL35	23	UL48A	U32	BFRF3	ORF65
portal protein	UL6	54	UL104	U76	BBRF1	ORF43
portal capping protein	UL25	34	UL77	U50	BVRF1	ORF19
Tegument and cytoplasmic egress						
virion protein kinase	UL13	47	UL97	U69	BGLF4	ORF36
largest tegument protein	UL36	22	UL48	U31	BPLF1	ORF64
LTP binding protein	UL37	21	UL47	U30	BOLF1	ORF63
encapsidation and egress protein	UL7	53	UL103	U75	BBRF2	ORF42
cytoplamsic egress tegumentprotein	UL11	49	UL99	U71	BBLF1	ORF38
CETP binding protein	UL16	44	UL94	U65	BGLF2	ORF33
cytoplasmic egress facilitator-1	UL51	7	UL71	U44	BSRF1	ORF55
encapsidation chaperoneprotein	UL14	46	UL95	U67	BGLF3	ORF34
capsid transport tegumentprotein	UL17	43	UL93	U64	BGLF1	ORF32
cytoplasmic egress facilitator-2	UL21	38	UL88	U59	BTRF1	ORF23
Putative membrane protein	UL24	35	UL76	U49	BXRF1	ORF20
Envelope						
glycoprotein B	UL27	31	UL55	U39	BALF4	ORF8
glycoprotein H	UL22	60	UL75	U48	BXLF2	ORF22
glycoprotein L	UL1	37	UL115	U82	BKRF2	ORF47
glycoprotein M	UL10	50	UL100	U72	BBRF3	ORF39
glycoprotein N	UL49A	9A	UL73	U46	BLRF1	ORF53
DNA Replication, recombination and metabolism						
DNA polymerase	UL30	28	UL54	U38	BALF5	ORF9
DNA polymerase processivitysubunit	UL42	16	UL44	U27	BMRF1	ORF59
helicase-primase ATPase subunit	UL5	55	UL105	U77	BBLF4	ORF44
helicase-primase RNA pol subunit B	UL52	6	UL70	U43	BSLF1	ORF56
helicase-primase subunit C	UL8	52	UL102	U74	BBLF2 BBLF3	ORF40 ORF41
single strand DNA binding protein	UL29	29	UL57	U41	BALF2	ORF6
alkaline deoxyribonuclease	UL12	48	UL98	U70	BGLF5	ORF37
deoxyuridinetriphosphatase	UL50	8	UL72	U45	BLLF3	ORF54
uracil-DNA glycosidase	UL2	59	UL114	U81	BKRF3	ORF46
ribonucleotide reductase large subunit	UL39	19	UL45	U28	BORF2	ORF61

Capsid assembly, DNA encapsidation and nuclear egress						
maturational protease	UL26	33	UL80	U53	BVRF2	ORF17
assembly protein	UL26.5 (UL26)	33.5 (33)	UL80.5 (UL80)	U53.5 (U53)	BdRF1 (BVRF2)	ORF17.5 (ORF17)
capsid transport nuclear protein	UL32	26	UL52	U36	BFLF1	ORF68
terminase ATPase subunit 1	UL15	42 45	UL89	U66	BGRF1 BDRF1	ORF29
terminase DNA binding subunit 2	UL28	30	UL56	U40	BALF3	ORF7
terminase binding protein	UL33	25	UL51	U35	BFRF1A	ORF67
nuclear egress membrane protein	UL34	24	UL50	U34	BFRF2	ORF66
nuclear egress lamina protein	UL31	27	UL53	U37	BFLF2	ORF69

1. Mocarski Jr ES. Comparative analysis of herpesvirus common proteins. In: Arvin A, Campadelli Fiume G, Mocarski E, Moore PS, Roizman B, Whitley R, Yamanishi K, editors. Human Herpesviruses: Biology, Therapy, and Immunoprophylaxis. Cambridge:Cambridge University Press; 2007.
