# Supplementary material for: Genome-wide analysis of G-quadruplexes in herpesvirus genomes
Source: BMC Genomics. 2016 Nov 21;17:949. doi: 10.1186/s12864-016-3282-1 (PMC5117502; doi:10.1186/s12864-016-3282-1)
Supplement: Additional file 4: Table S3. — Temporally regulated genes. List of herpesvirus genes categorized as immediate early, early and late genes. (PDF 89 kb) [file 12864_2016_3282_MOESM4_ESM.pdf]

Table S3. List of herpesvirus genes categorized as immediate early (IE), early (E) and late(L) genes.

Virus name	Temporal classification	Gene names
**HHV-1/2**	**IE**	RL2, UL54, RS1, US1, US12
	**E**	UL2, UL9, UL8 , UL5, UL12, UL23, UL29, UL30, UL39, UL40, UL42, UL52, UL50
	**L**	UL3, UL6, UL10, UL11, UL13, UL14, UL15, UL16, UL17, UL18, UL19, UL20, UL21, UL22, UL24, UL25, UL31, UL32, UL33, UL34, UL35, UL37, UL38, UL41, UL42, UL43, UL44, UL45, UL51, UL53, UL56, US5, UL36, UL49A, US11, UL4, US2, UL55, UL26, UL48, UL6, US9, UL26.5, UL28
**HHV-3**	**IE**	ORF4, ORF63, ORF62, ORF61
	**E**	ORF16, ORF18, ORF19, ORF28, ORF29, ORF36, ORF48, ORF51, ORF52, ORF55, ORF59, ORF6, ORF8
	**L**	ORF14, ORF15, ORF16, ORF18, ORF20, ORF21, ORF23, ORF24, ORF25, ORF26, ORF27, ORF34, ORF35, ORF37, ORF38, ORF39, ORF40, ORF41, ORF42, ORF43, ORF44, ORF46, ORF47, ORF50, ORF54, ORF58
**HHV-4**	**IE**	BZLF1, BRLF1, BMLF1
	**E**	BALF1, BALF2, BALF5, BaRF1, BARF1, BBLF2/3, BBLF4, BFRF1, BGLF4, BGLF5, BHLF1, BHLF2, BHRF1, BKRF3, BLLF3, BMLF1, BMRF1, BNLF2a, BORF2, BRRF1, BSLF1, BSLF2, BXLF1
	**L**	BALF3, BALF4, BBLF1, BBRF1, BBRF3, BCRF1, BDLF1, BDLF2, BDLF3, BdRF1, BFRF3, BGLF1, BGLF2, BILF1, BILF2, BKRF2, BKRF4, BLLF1, BLRF1, BLRF2, BMRF2, BOLF1, BORF1, BPLF1, BRRF2, BSRF, BVRF2, BXLF2, BZLF2
**HHV-5**	**IE**	UL110, UL122, UL123, US3, UL36, UL37, UL38, IRS1
	**E**	IRL2, IRL7, IRL14, UL4, UL5, UL13, UL17, UL26, UL27, UL33, UL35, UL36, UL53, UL54, UL55, UL56, UL57, UL58, UL63, UL104, UL105, UL112, UL114, UL118, UL119, UL124, UL128, US8, US9, US10, US11, US12, US13, US14, US16, US17, US18, US19, US20, US21, US22 , UL77, UL78, US23, US24, US26 US27, US28, US30, US33, US34
	**L**	IRL3, TRL140, UL2, UL3, UL7 , UL9, UL14, UL15, UL18, UL21, UL29, UL31, UL32, U41, UL43 , UL48, UL52, UL59, UL60, UL67, UL68, UL91, UL92, UL93, UL94, UL102, UL103, UL107, UL108, UL109, UL111, UL120, UL121, UL129, UL131, UL81, UL82, US32
**HHV-8**	**IE**	ORF11, ORFK4, ORFK4.1, ORFK5, PAN, ORF16, ORF45, ORF50
	**E**	ORF6, ORF17.5, ORF18, ORF34, ORF35, ORF36, ORF37, ORF38, ORF39, ORF46, ORF47, ORF58, ORF59, ORF60, ORF61, ORFK3, ORFK14, ORF74
	**L**	ORF8, ORF9, ORF10, ORFK3A, K5/6, ORF17, ORF21, ORF22, ORF23, ORF24, ORF25, ORF26, ORF27, ORF28, ORF30, ORF31, ORF33, ORF40/41, ORF42, ORF44, ORF52, ORF53, ORF55, ORF56, ORFK9 , ORFK10, ORFK10.5, ORFK11, ORF65, ORF66, ORF67 ORF67.5 ,ORF69, ORF75, K8.1
