# Supplementary material for: Genome-wide analysis of G-quadruplexes in herpesvirus genomes
Source: BMC Genomics. 2016 Nov 21;17:949. doi: 10.1186/s12864-016-3282-1 (PMC5117502; doi:10.1186/s12864-016-3282-1)
Supplement: Additional file 5: Table S4. — PQS oligonucleotides. Names and sequences of virus PQS oligonucleotides randomly selected for CD spectroscopy. (PDF 92 kb) [file 12864_2016_3282_MOESM5_ESM.pdf]

Table S4. Names and sequences of virus PQS oligonucleotides randomly selected for CD spectroscopy.

Oligonucleotide name	Oligonucleotide sequence	Virus	Nucleotide Position
O1	GGGGGGGGGGGCGCGCCGGGGCTCCTGGGG*	HHV-8	113142
O2	GGGATGACGCGGGCCCCGGGCAGGG	HHV-2	148846
O3	GGGTGGGGGGGCCTGGG	HHV-1	91210
O4	GGGCAGGAAGGGAAAGCCCGGGACATAGGG	HHV-4	105821
O5	GGGGCCGGGGATGGGGGGGAAGGG	HHV-2	5684
O6	GGGTCGGGTGGGACGTGCGGG*	HHV-8	37802
O7	GGGGCCGGGGAGGGCTGGGGCCGGGG	HHV-1	151537
O8	GGGGACGGGGCCTTCTGGGGAAAATGGGG	HHV-8	93209
O9	GGGGAGGGACGGGGAAGGGGGCGCGCGGGG	HHV-2	123
O10	GGGGGGGGGGGGGGGGGG	HHV-1	8931
O11	GGGGGGAGAGGGGAGAGGGGGGGAGAGGGG	HHV-1	9098
O12	GGGGCGGGAGGGGGCGAGGGGCGGGAGGGG	HHV-1	151094
O13	GGGGGGCCGGGGGGCCGGGGGGCCGGGGGG	HHV-2	133385
O14	GGGGGGACGGGGGGACGGGGGGACGGGGGG	HHV-2	133417
O15	GGGGTAGGGAGGGATGAAGGG*	HHV-6B	154601
ssDNA	GCTATGGCTTGCTATGGCTTGCTAT	-	-
c-myc	TGGGGAGGGTGGGGAGGGTGGGGAAGG	-	-
G-rich (-) control	GGTGTGTGTGTGTGTGTGTGTG	-	-
Hybrid PQS control	TTAGGGTTAGGGTTAGGGTTAGGGTT	-	-

* Denotes PQS in the reverse complement of the genome.
