# Supplementary material for: Genome-wide analysis of G-quadruplexes in herpesvirus genomes
Source: BMC Genomics. 2016 Nov 21;17:949. doi: 10.1186/s12864-016-3282-1 (PMC5117502; doi:10.1186/s12864-016-3282-1)
Supplement: Additional file 7: Table S5. — Promoter PQS oligonucleotides. Sequence of PQS oligonucleotides from the promoter region of UL24, UL2, and K15. (PDF 82 kb) [file 12864_2016_3282_MOESM7_ESM.pdf]

Table S5. Sequence of PQS oligos from the promoter region of UL24, UL2, and K15.

PQS name	Sequence
UL24	GGGTGAGGGCCGGGGGCGGGG
UL2	GGGGATTTTGGGTTGGGTCGGG
K15	GGGGTCCCCCGCGGGCGGGGGCGGGG
