# Supplementary material for: Genome-wide analysis of G-quadruplexes in herpesvirus genomes
Source: BMC Genomics. 2016 Nov 21;17:949. doi: 10.1186/s12864-016-3282-1 (PMC5117502; doi:10.1186/s12864-016-3282-1)
Supplement: Additional file 9: — Primer sequences. Primer sequences used in this study. (PDF 149 kb) [file 12864_2016_3282_MOESM9_ESM.pdf]

Table S6. Primer sequences:

**Name**	**Nucleotide positions***	**Sequence (5'->3')****
UL24 F.P	47237-47258	AT*GGTACC*GCCAGCACGGCCTGGGGGGTCA
UL24 R.P	47722-47702	AT*AAGCTT*GGCGTTGCGCCCTCGCCGGCA
UL2 F.P	9325-9343	AT*GGTACCA*CCCTAGGCGCTATGGGGA
UL2 R.P	9834-9814	AT*GAGCTC*CAAATCCTGTCGCCCTACACA
K15 F.P	137505-137489	AT*GGTACC*TGTTCACGTAGTGTCCA
K15 R.P	137081-137098	AT*GAGCTC*ACTAGGTATCCACAGGGC
ORF50 F.P	71112-71130	AT*CTCGAG*GATGTGGTACCGAATGCCA
ORF 50 R.P	71412-71394	AT*AAGCTT*TTTTTGTGGCTGCCTGGAC

* Nucleotide positions are with reference to accession number X14112 for UL2 and UL24 and accession number GQ994935.1 for K15 and ORF 50.
** The restriction enzyme sites are italicized.
